# *Withania somnifera* Root Extract Enhances Chemotherapy through ‘Priming’

**DOI:** 10.1371/journal.pone.0170917

**Published:** 2017-01-27

**Authors:** Aine Brigette Henley, Ling Yang, Kun-Lin Chuang, Meliz Sahuri-Arisoylu, Li-Hong Wu, S. W. Annie Bligh, Jimmy David Bell

**Affiliations:** 1 Department of Life Sciences, Faculty of Science and Technology, University of Westminster, London, United Kingdom; 2 Department of Chinese Pharmaceutical Sciences and Chinese Medicine Resources, College of Pharmacy, China Medical University, Taichung, Taiwan; 3 Institute of Chinese Materia Medica, Shanghai University of Traditional Chinese Medicine, Shanghai, People's Republic of China; Columbia University, UNITED STATES

## Abstract

*Withania somnifera* extracts are known for their anti-cancerous, anti-inflammatory and antioxidative properties. One of their mechanisms of actions is to modulate mitochondrial function through increasing oxidative stress. Recently ‘priming’ has been suggested as a potential mechanism for enhancing cancer cell death. In this study we demonstrate that ‘priming’, in HT-29 colon cells, with *W*. *somnifera* root extract increased the potency of the chemotherapeutic agent cisplatin. We have also showed the *W*. *somnifera* root extract enhanced mitochondrial dysfunction and that the underlying mechanism of ‘priming’ was selectively through increased ROS. Moreover, we showed that this effect was not seen in non-cancerous cells.

## Introduction

Cancer is one of the major causes of death around the globe, despite the progress observed in surgery, radiation and chemotherapy [1–3]. Cancer progression relies on the ability of cancer cells to exploit the normal physiological processes of the host [1]. This unfortunately means that the cytotoxicity of chemotherapeutic drugs is not limited to cancer cells and can affect non-cancer cells [1, 4]. Today the most common therapeutic strategy of chemotherapy drugs is the use of two drugs in combination, however this is invariably associated with side effects which include chemoresistance [5–7]. It is therefore essential to explore and enhance current methods of chemotherapy to improve their efficacy while also reducing side effects. One such approach is ‘priming’, whereby cancer cells are pre-treated with a ‘priming’ agent (curcumin, quercetin, aspirin) prior to chemotherapy treatment [8–12]. The underlying mechanism underpinning ‘priming’ appears to be the enhancement of cell death through mitochondrial dysfunction [8, 9]. Mitochondrial dysfunction can alter ROS levels, ATP production and overall cell viability and is a novel key target in cancer treatment [9, 13, 14].

*Withania somnifera* is an Ayurvedic medicinal plant whose root and leaves extracts have been used for its antioxidant and restorative properties as well as to reduce cancer growth [2, 6, 15]. *W*. *somnifera* extracts have been found to be effective in treating several types of cancer including skin, leukaemia, breast, colon and pancreas [15–21]. However, the mechanisms of action have yet to be fully elucidated, but indications of involvement in mitochondrial membrane permeability have been reported in several studies [1, 21–23]. Additionally, *W*. *somnifera* extracts have been shown to increase reactive oxygen species (ROS) [1, 19, 23]. The mitochondria is an important regulator of cell survival and progression and is the main source of ROS which is linked with mitochondrial function [24]. Cancer cells metabolism is known to have an altered phenotype whereby they primarily respire through lactate production in a process known as the ‘Warburg Effect’ [25–27]. This alteration in metabolism is a key hallmark of cancer cell progression and has been linked to an alteration in mitochondria function [9, 25]. *W*. *somnifera* has been reported to induce mitochondrial dysfunction in human leukaemia cells and also reduce mitochondrial function in breast cancer cells [1, 19]. Investigating this mitochondrial alteration further could highlight the value of *W*. *somnifera* as an anti-tumour agent.

Mitochondrial dysfunction can alter ROS levels, ATP production and overall cell viability and is a novel key target in cancer treatment [9, 13, 14]. In this study we investigated the potential of *W*. *somnifera* as a ‘priming’ agent, and showed that ‘priming’ with this root extract enhanced the efficacy of cisplatin through increased ROS in cancer cells while having no detectable effect on non-cancer cells.

## Materials and Methods

### Extraction

The extraction method was performed according to the British Pharmacopeia. *Withania somnifera* root powder (1.0 g, Lot no. 6051SS/03, Pukka, UK), was shaken with 2 mL of dilute ammonia R4. Methanol (20 mL) was added and the mixture was sonicated for 20 minutes. It was then heated on the water bath for 3 minutes and filtered. The filtrate was evaporated to dryness at 60°C. A stock solution of 0.08335 g of dry extract /mL DMSO was prepared for biological studies.

### HPTLC

The dry extract was reconstituted in methanol and filtered. The methanolic extract was then applied to a precoated silica gel 60 F_254_ high performance plate (Merck). CAMAG HPTLC System (Automatic TLC Sampler 4; ADC2 Automatic Developing Chamber; TLC Visualizer; Chromatogram Immersion Device III; TLC Plate Heater III; VisionCats software) was used. Application: 2 μL of reference and test solutions. Mobile phase: Toluene, ethyl acetate, formic acid 10:3:1 (v/v/v). Derivatization: 5% sulphuric acid methanol. Dip (time 0, speed 5), heat at 110°C for 2 min, detection at UV 366 nm.

### HPLC

The methanolic root extract (0.3 mg/mL) was analysed using Waters ACQUTTY UPLC—Synapt G2 QTOF (Waters, USA) and a BEH C18 (2.1×100 mm, 1.7 μm) column (Waters, USA) at 40°C to provide efficiency to the peaks. The mobile phase consisted of water (A) and methanol (B), which were applied in the following gradient elution: from 60:40 (A:B) in 15 min to 20:80(A:B). The flow rate and sample volume were set to 0.3 mL/min. The sample volume was 10 μL. The ESI source was operated in positive (ESI+) ionization mode. The optimized conditions to trigger maximum response of metabolites were listed as follows: capillary voltage, +3 kV; sample cone, + 30 V; extraction cone, +4.0 V; source temperature, 120°C; desolvation temperature, 350°C; cone gas (nitrogen) flow, 50 L/h; and desolvation gas (nitrogen) flow, 600 L/h. Argon was used as collision gas. Leucine- enkephalin (2 ng/mL) was used as the lock mass generating a reference ion at m/z of 556.2771 by a lockspray at 5 μL/min to acquire accurate mass during analysis.

### Cell culture

Human breast cancer cell line MDA-MB231 and HT-29 colon cancer cells were grown in DMEM (Sigma, UK) with 10% FBS (Sigma), 2% L-Glutamine (Invitrogen, UK) and 2% Penicillin/Streptomycin (P/S) solution (Invitrogen). MDA-MB231 cells were obtained from Dr. T. Kalber (Medical Research Council Clinical Sciences Centre, London) and HT-29 cells were donated by Dr. N. Haiji (Imperial College London). Human non-cancer breast epithelial MCF10A cells were grown in DMEM:F12 (Life Sciences, UK) supplemented with 5% horse serum (Sigma), 2% P/S, 20 ng/mL epidermal growth factor (Sigma), 0.5 mg/mL hydrocortisone (Sigma), 100 ng/mL cholera toxin (Sigma), 10 μg/mL insulin (Sigma). MCF10A cells were donated by Dr. N. Haiji (Imperial College London). All cells were maintained at 37°C in a humidified 5% CO_2_ atmosphere.

### Treatment conditions

All cells were treated with *W*. *somnifera* extract (0 μg/mL -10 μg/mL) for 48 h prior to assessment. Primed cells were treated with *W*. *somnifera* extract (0 μg/mL -10 μg/mL) for 48 h, washed with PBS and incubated with 100 μM Cisplatin for a further 24 h. ‘Priming’ with quercetin was carried out as follows with 24 h of 40 μM quercetin followed by 100 μM cisplatin for 24 h [11, 28].

### 3-(4,5-dimethylthiazol-2-yl)-2,5-diphenyl-tetrazolium bromide (MTT) assay

Cell viability was determined using a colorimetric 3-(4,5-dimethylthiazol-2-yl)-2,5-diphenyl-tetrazolium bromide (MTT) assay (Sigma, UK). All cells were seeded in a 96-well plate at a density between 1.5–3 x10^4^ cells per well and were treated with the various treatment conditions as described above. Cell viability was assayed according to the manufacturers’ protocol. Absorbance was determined at 570 nm and normalised with 690 nm background with a micro plate reader (Spectramax 340PC) after 3.25 h. The optical density was measured as a percentage of the control.

### ROS assay

Cellular ROS was measured using a 2’,7’–dichlorofluorescein diacetate (DCFDA) Assay (Abcam, UK). The fluorescence was detected on a fluorescent plate reader (FLUOstar Omega, BMG Labtech, UK) with an excitation of 495 nm and emission of 529 nm. Cells were seeded at 2 x10^4^ in 96-well black bottom plate. DCFDA incubation was carried out according to the protocol. Cells were treated using the various treatment conditions previously described. The optical density was measured as a percentage of the control and normalised to cell count.

### ATP production, proton leak and mitochondrial respiration

ATP, proton leak and mitochondrial/non-mitochondrial respiration was measured using a Seahorse Bioanalyser (Seahorse Biosciences, USA). MDA-MB231, HT-29 and MCF10A cells were seeded 1–2.5 x10^4^ cells per well in a specific Seahorse Bioanalyser 24 well plate and treated appropriately as previously described. The protocol was carried out as specified by the manufacturer’s instructions. Oligomycin (1 μg), carbonyl cyanide-4-(trifluoromethoxy)phenylhydrazone (FCCP) (0.2 μM: MDA-MB231, 0.4 μM: MCF10A, 0.6 μM: HT-29) and antimycin/rotenone (0.25 μM) were added to the sensor plate in the appropriate dilutions directly prior to the commencement of the calibration and assay. Calculations were normalised to protein level using a standard Bradford assay (Biorad).

### Assessment of drug-drug interactions

To assess the effects of the combination of *W*. *somnifera* and cisplatin synergy or antagonism was calculated according to the methods described by Prichard and Shipman [29]. The raw data of 5 experiments was analysed at the 95% confidence interval using the MacSynergy II software developed by Prichard and Shipman. The program calculates the synergy or antagonism of the drug interactions by calculating theoretical additive interactions from the dose-response surface.

### Statistical analysis

Statistical analysis was carried out using a one-way ANOVA with Tukey correction and one sample t-test on Graphpad, Prism Software. Results are presented as mean ± SEM. Significance taken when p<0.05.

## Results

### Cell viability assessment of *W*. *somnifera* root extract

**Fig 1:**
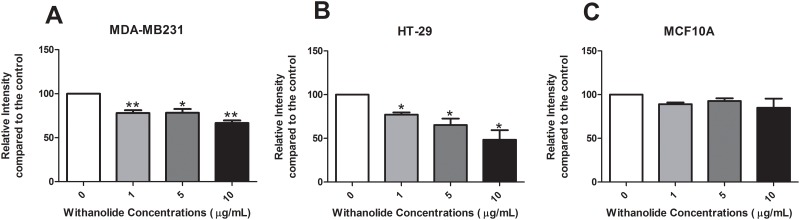
Assessing cell viability of *W*. *somnifera* root extract on cancer and non-cancer cells. Cells were treated with increasing concentrations (1 μg/mL *-*10 μg/mL) of *W*. *somnifera* root extract for 48 h in **(A)** MDA-MB231, **(B)** HT-29 and **(C)** MCF10A cells. Data represent the average of 5 independent experiments ± SEM. *p<0.05, **p<0.01 *vs* non-treatment (0).

The direct effect of treating cancer cells with *W*. *somnifera* root extract was examined using an MTT assay. Cancer and non-cancer cells were treated with a titration of 1 μg/mL, 5 μg/mL and 10 μg/mL *W*. *somnifera* for 48 h. A significant reduction of cell viability was observed in breast (MDA-MB231) cancer cells compared to non-treatment in a dose dependant manner (p<0.01) (Fig 1A). Similar effect was observed in HT-29 colon cancer cells (p<0.05) (Fig 1B). However, in non-cancer cells (MCF10A) there was no reduction in cell viability compared to non-treatment (Fig 1C).

### The effect of *W*. *somnifera* extract on oxidative stress

**Fig 2:**
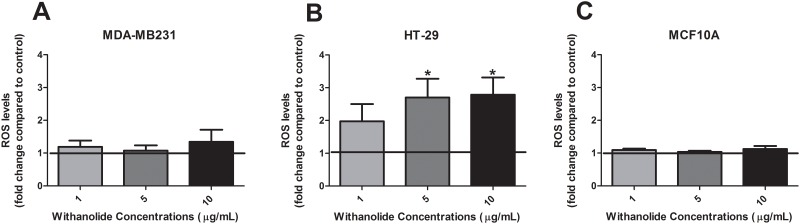
Oxidative stress alteration following treatment with *W*. *somnifera* in cancer and non-cancer cells. ROS levels were measured using a DCFDA assay following 48 h treatment with 1 μg/mL, 5 μg/mL and 10 μg/mL of *W*. *somnifera* root extract in **(A)** MDA-MB231, **(B)** HT-29 and **(C)** MCF10A cells. The bold line in each graph represents control treatment levels for the experiment. Data represent the average of 5 independent experiments ± SEM. *p<0.05 *vs* non-treatment.

Oxidative stress is an important marker of cellular response and can indicate early signs of cell death. There were no changes in ROS levels following treatment with *W*. *somnifera* alone in MDA-MB231 cancer cells or MCF10A non-cancer cells (Fig 2A and 2C). However, in HT-29 cancer cells there was a significant increase in ROS levels compared to the control following 48 h of treatment with 5 μg/mL and 10 μg/mL of *W*. *somnifera* root extract (p<0.05) (Fig 2B).

### Mitochondrial function following *W*. *somnifera* treatment

**Fig 3:**
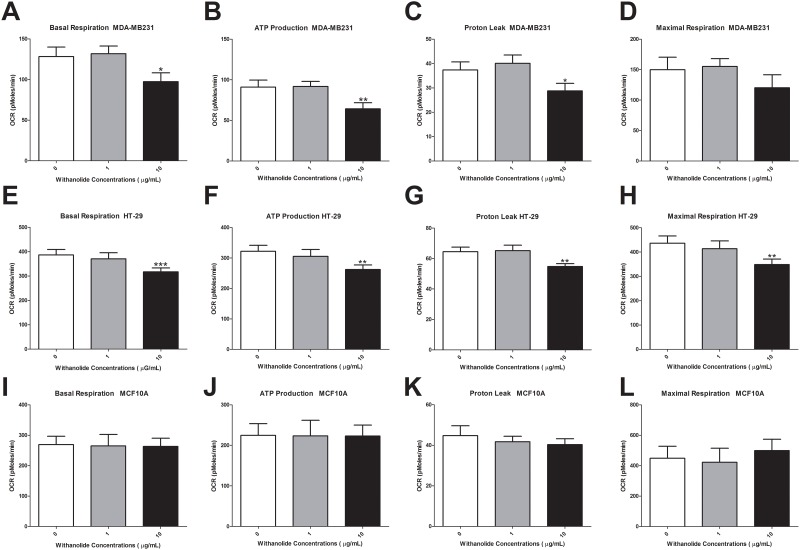
Mitochondrial functional analysis following 48 h of *W*. *somnifera* root extract treatment in MDA-MB231, HT-29 and MCF10A cells. Mitochondrial function was assessed using a Seahorse Bioanalyser following 48 h of 1 μg/mL and 10 μg/mL *W*. *somnifera* root extract treatment. Alterations in MDA-MB231 cancer cells **(A)** basal respiration, **(B)** ATP production, **(C)** proton leak and **(D)** maximal respiration was investigated following treatment. The same functional alterations were observed in HT-29 cells (E–H) and in MCF10A cells (I-L). Data represents the average of 3 independent experiments ± SEM. *p<0.05, ** p<0.01, *** p<0.001 *vs* non-treatment.

Mitochondrial basal respiration of MDA-MB231 cells was reduced following 48 h of treatment with 10 μg/mL *W*. *somnifera* root extract (p<0.05) (Fig 3A). The cell’s ATP production levels (p<0.01) and proton leak (p<0.05) were also reduced (Fig 3B, 3C and 3D). However, there was no change in maximal respiration. Interestingly in HT-29 cells this reduction in mitochondrial function was more pronounced for basal respiration (p<0.001), ATP production levels (p<0.01), proton leak (p<0.01) and maximal respiration (p<0.01) (Fig 3E, 3F, 3G and 3H). The mitochondrial function in non-cancer MCF10A cells was unchanged following *W*. *somnifera* root extract treatment (Fig 3I, 3J, 3K and 3L).

### ‘Priming’ with *W*. *somnifera* root extract enhances chemotherapy

**Fig 4:**
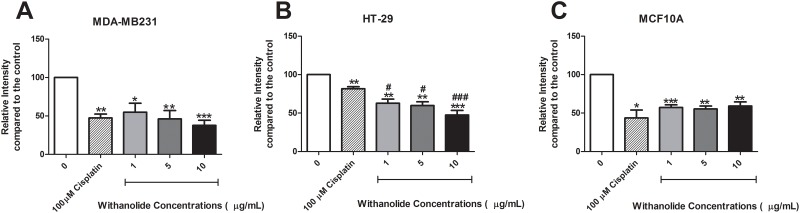
The effect of ‘priming’ with *W*. *somnifera* root extract prior to cisplatin treatment on cell viability. Cell viability was assessed following ‘priming’ with *W*. *somnifera* root extract (1 μg/mL, 5 μg/mL and 10 μg/mL) for 48 h prior to 100 μM cisplatin treatment. **(A)** MDA-MB231, **(B)** HT-29 and **(C)** MCF10A cell viability following ‘priming’ with *W*. *somnifera* root extract and 100 μM cisplatin treatment alone. Data represents the average of 5 independent experiments ± SEM. *p<0.05, ** p<0.01, *** p<0.001 *vs* non-treatment, # p<0.05, ### p<0.001 *vs* cisplatin.

Cisplatin treatment reduced cancer cell growth in MDA-MB231 cells (p<0.01), HT-29 (p<0.01) and MCF10A cells (p<0.05) (Fig 4). The drug combinations of cisplatin and *W*. *somnifera* were assessed to observe if their activities were synergistic or antagonistic following the Prichard and Shipman equation [29]. The drug-drug interactions were analysed at a 95% confidence limit and displayed as peaks above (synergy) and below (antagonism) a predicted additive plane in a 3-D graph (S3 Fig). MDA-MB231, HT-29 and MCF10A cells were antagonistic following combination of cisplatin and *W*. *somnifera*.

‘Priming’ with *W*. *somnifera* (treatment: 48 h prior to 100 μM cisplatin) showed there was no added effect in MDA-MB231 cells (Fig 4A). However, in HT-29 colon cancer cells ‘priming’ with 1 μg/mL of *W*. *somnifera* root extract showed an added effect compared to cisplatin treatment alone (p<0.05) (Fig 4B). This enhanced effect increased with increasing *W*. *somnifera* root extract concentration (p<0.001). There was no enhanced effect of ‘priming’ in non-cancer MCF10A cells (Fig 4C). Indeed, there was a slight trend to increased cell viability compared to cisplatin treatment though this did not reach significance.

As a positive control of ‘priming’ the cells were treated with quercetin, an agent that has been shown to enhance chemotherapy following this ‘priming’ method [11, 28]. Following the same incubation conditions MDA-MB231 showed a reduction in cell viability following ‘priming’ with quercetin though this was not enhanced compared to cisplatin treated alone (Fig A in S4 Fig). This effect was also observed with HT-29 and MCF10A cells (Figs B and C in S4 Fig).

### ROS is key in establishing the effect of ‘priming’ with *W*. *somnifera* root extract

**Fig 5:**
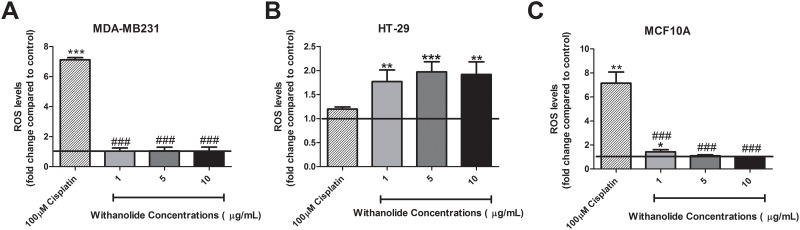
Oxidative stress response following ‘priming’ with *W*. *somnifera* root extract. The alteration to oxidative stress levels was assessed following ‘priming’ with *W*. *somnifera* from 1 μg/mL to 10 μg/mL 48 h prior to 100 μM cisplatin treatment in **(A)** MDA-MB231, **(B)** HT-29 and **(C)** MCF10A cells. The bold line in each graph represents control treatment levels for the experiment Data represents the average of 5 independent experiments ± SEM. *p<0.05, **p<0.01, ***p<0.001 *vs* non-treatment, # p<0.05, ## p<0.01, ### p<0.001 *vs* cisplatin.

When treated with cisplatin there was an increase in ROS levels in MDA-MB231 (p<0.001) HT-29 (p<0.01) and MCF10A (p<0.01) cells (Fig 5).

Interestingly ‘priming’ with *W*. *somnifera* reduced the effect cisplatin had on ROS levels in MDA-MB231 cancer cells (p<0.001), bringing it to a sustainable level (Fig 5A). However, in HT-29 cells an increase in ROS levels were observed following ‘priming’ compared to cisplatin treatment alone (p<0.05). In non-cancer MCF10A cells following ‘priming’, a reduction of ROS levels was observed compared to cisplatin treatment (p<0.001) (Fig 5C).

### ‘Priming’ with *W*. *somnifera* root extract increases mitochondrial dysfunction

**Fig 6:**
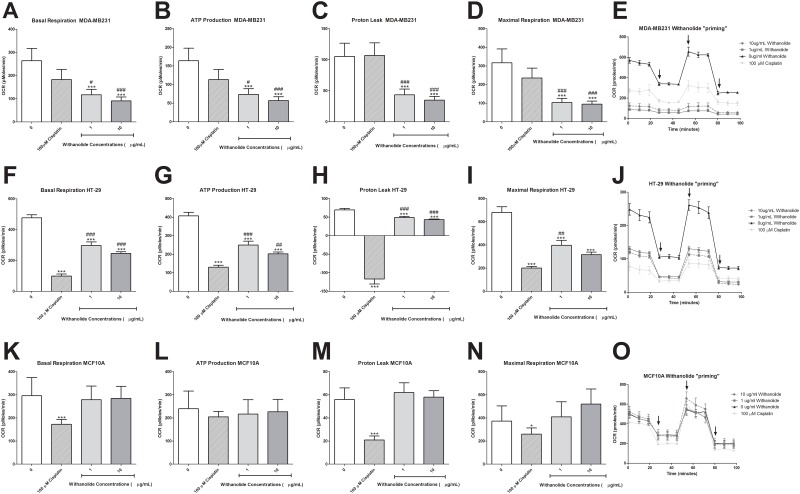
‘Priming’ with *W*. *somnifera* root extract alters mitochondrial function. Mitochondrial function was analysed using the Seahorse Bioanalyser to observe if there was any change following ‘priming’. MDA-MB231 **(A)** basal respiration, **(B)** ATP production, **(C)** proton leak and **(D)** maximal respiration was analysed following ‘priming’ with *W*. *somnifera* root extract. **(E)** A representative graph of the experiment shows the experimental procedure and injection set up with the first arrow representing oligomycin, second arrow FCCP and third arrow representing antimycin and rotenone. HT-29 cells **(F-J)** and MCF10A cells **(K-O)** were also assessed. Data represents the average of 3 independent experiments ± SEM. *p<0.05, ** p<0.01, *** p<0.001 *vs* non-treatment, # p<0.05, ## p<0.01, ### p<0.001 *vs* cisplatin.

There was no effect of cisplatin treatment on mitochondrial function in MDA-MB231 cells only a slight trend to decrease in basal respiration, ATP production and maximal respiration (Fig 6A, 6B, 6C and 6D). However, the HT-29 cells basal mitochondrial respiration was decreased following cisplatin treatment (p<0.001) along with ATP production levels (p<0.001), proton leak (p<0.001) and maximal respiration (p<0.001) (Fig 6F, 6G, 6H and 6I). Interestingly MCF10A cells also had a reduction in basal respiration following cisplatin treatment (p<0.001) (Fig 6K). There was also a reduced change in proton leak (p<0.001) and maximal respiration (p<0.05) (Fig 6M and 6N), but not in ATP levels.

‘Priming’ in MDA-MB231 reduced basal respiration rate (p<0.001), ATP production (p<0.001), proton leak (p<0.001) and maximal respiration rate (p<0.001) (Fig 6A, 6B, 6C and 6D). Similarly, HT-29 cells following ‘priming’ decreased basal respiration (p<0.001), ATP production levels (p<0.001), proton leak (p<0.001) and maximal respiration (p<0.001) (Fig 6F, 6G, 6H and 6I). However, the reduction observed following ‘priming’ in HT-29 cells was not as reduced as cisplatin treatment alone and the differences observed were significantly different; basal respiration (p<0.001), ATP production (p<0.001), proton leak (p<0.001) and maximal respiration (p<0.01). ‘priming’ reversed the effects of cisplatin on MCF10A cells mitochondrial function (Fig 6K, 6L, 6M and 6N).

## Discussion

This study has shown that ‘priming’ with *W*. *somnifera* root extract leads to enhanced cell death in HT-29 colon cancer cells but not in MDA-MB231 cells or MCF10A non-cancer cells. We propose that the ‘priming’ effect of *W*. *somnifera* root extract was exclusive to HT-29 cancer cells due to increased ROS production and reduced mitochondrial function.

*W*. *somnifera* has many positive medicinal properties which include antitumour, antioxidative and anti-inflammatory effects [1, 19, 20, 30]. The efficacy of Withaferin A in *W*. *somnifera* has been shown to have an effect on killing breast cancer cells from 1 μM to 5 μM (17,29). Interestingly, in this study using a lower dose of 1 μg/mL *W*. *somnifera* root extract for 48 h showed a reduction in breast cancer cells and colon cancer cells but had no effect on non-cancer cells. This suggests that lowering the concentration but prolonging the treatment period could have an even greater effect on cancer cell inhibition without effecting normal cells. It has been reported that using 2.5 μM Withaferin A for 6 h can increase oxidative stress in MDA-MB231 cells [19]. However, using *W*. *somnifera* root extract for 48 h had no effect on oxidative stress in MDA-MB231 cells. There was an increase in oxidative stress in HT-29 cells which proposes that breast cancer cells are able to protect themselves from *W*. *somnifera* treatment over a prolonged period.

Although the use of chemotherapeutic agents in killing cancer cells is a very effective treatment, it can lead to several side effects which include chemoresistance [31–33]. An emerging new method of cancer treatment is ‘priming’ which sensitizes cells to chemotherapy treatment [8–10, 28]. This technique has most potential when looking at aggressive tumours such as triple negative breast cancer, MDA-MB231, which have the worst outcome after chemotherapy than any other breast cancer subtype [34, 35]. Triple negative breast cancers account for 20–25% of all breast cancer cases [36]. The response of this subgroup of breast cancer to treatment relies heavily on chemotherapy as they do not respond to endocrine therapy or trastuzumab [35, 36]. Another tumour that is difficult to treat is the clinically diverse colon cancer which is difficult to treat due to its heterogeneity [37]. Along with its heterogeneity is its poor response to drug treatments which could be in part due to underlying molecular changes altering its genetic stability [38, 39] One such study identified, BRAF inhibition by the small-molecule drug PLX4032 is extremely effective in melanoma treatment but colon cancer cells with the same BRAF lesions do not respond to the same drug [39]. This poor response to drug treatments highlights the importance of developing novel therapeutic treatments. Using this new approach, we showed that ‘priming’ with *W*. *somnifera* root extract enhanced the therapeutic effect of cisplatin in HT-29 cells. Furthermore, we showed that there was no added effect in non-cancer cells.

A key characteristic of cancer cells is an alteration in metabolism to a process known as the ‘Warburg Effect’ [27, 40]. In this process, cancer cells respiration is primarily anaerobic being driven through lactate production rather than through the aerobic citric acid cycle. One key mechanism of ‘priming’ is mitochondrial dysfunction whereby mitochondrial respiration is reduced due to increased VDAC1 activity and increased oxidative stress [4, 8, 9, 41, 42]. ROS are important constitutively expressed regulators that control both cell survival and cell death [24, 43, 44]. The main source of cellular ROS is from the mitochondria which is also a prime target when excessive ROS is expressed [19, 24, 43, 45, 46]. Uncontrolled levels of ROS can increase cell death through mitochondrial dysfunction [24, 43, 45, 46]. ‘Priming’ with *W*. *somnifera* root extract in HT-29 cells increased ROS levels compared to control while also enhancing cell death compared to cisplatin treatment alone. Furthermore, in HT-29 cells there was a reduction in basal mitochondrial respiration, ATP production and maximal respiration suggesting reduced mitochondrial function. The interaction between ATP production and ROS is tightly linked and abnormal activity can lead to TCA cycle damage [14, 23].This mitochondrial profile was replicated with MDA-MB231 cells however there was no change in ROS suggesting that even though the mitochondrial function is reduced there was no added oxidative stress enhancing the effect of cisplatin. There is no added generation of ROS from the mitochondria in the MDA-MB231 cells following ‘priming’ which is key in opening the mitochondrial membrane and activating cell death [14, 47]. More importantly there was no evidence of added cell death with non-cancer cells, no added ROS concentrations and no alteration to mitochondrial respiration. There was a slight trend to increase in cell viability and maximal respiration rate suggesting the non-cancer cells were increasing their cell growth in response to ‘priming’ with *W*. *somnifera* root extract.

Interestingly, both MDA-MB231 and HT-29 cells have mutant p53 (42). However, the mutation present in HT-29 cells, R273H, has been identified to increase drug resistance, proliferation and avoidance of cell death (43). This dominant-negative mutation is one of the most prevalent p53 mutations and is not evident in MDA-MB231 cells (44). This mutation can mask wild-type p53 in the cell to abrogate its function which is crucial in oxidative phosphorylation (27, T44). The reduction in mitochondrial function following ‘priming’ in MDA-MB231 cells was more enhanced than cisplatin alone. This effect was not as pronounced in HT-29 cells which could be due to the restoration of the wild-type p53, leading to increased cell death from ‘priming’ (45). This could explain the difference in mitochondrial function observed between MDA-MB231 and HT-29 cells whereby an effect in cell death was observed in the latter cell type.

To conclude, *W*. *somnifera* root extract enhances the effect of cisplatin in killing HT-29 cells through increasing ROS and mitochondrial dysfunction. This effect appears to be mainly through increased oxidative stress as there was no ‘priming’ effect in MDA-MB231 cancer cells. It has been suggested that mitochondria are the gatekeepers to chemotherapy and are ideal therapeutic targets for cancer therapy, therefore the impact of *W*. *somnifera* in enhancing chemotherapy through mitochondrial dysfunction may prove an important new approach. Further studies are required to examine the molecular mechanism of ‘priming’ with *W*. *somnifera* root extract.

## Supporting Information

S1 Fig HPTLC. HPTLC image graphs of the extract samples. Graph A is measured at the wavelength 366 nm and B is the same graph under white RT light. BP means the extraction method is followed by British Pharmacopoeia. Two replications of each extract samples. The Rf value represents 0.09 (Withaferin A), 0.014 (Standard/Withanolide A) 0.24 (Withanolide B),0.6 (β-sitosteol).
(PDF)

S2 Fig HPLC-MS/MS data. **(A)** Ashwagandha root extract total ion chromatogram, **(B)** Ashwagandha root extract 471 [M+H]^+^ ion chromatogram, **(C)** Withanolide, peak 1 mass spectrum, **(D)** Withanolide, peak 2 mass spectrum, **(E)** Withanolide, peak 3 mass spectrum.
(PDF)

S3 Fig Evaluating the drug-drug interactions upon combining treatments of *W*. *somnifera* and cisplatin. The interactions of *W*. *somnifera* and cisplatin was assessed by the methods described in Prichard and Shipman (29). Cell viability was examined following treatment with *W*. *somnifera* root extract (0 μg/mL -10 μg/mL) in combination with cisplatin (0 μM -150 μM). **(A)** MDA-MB231, **(B)** HT-29 **(C)** MCF10A 3D models and data table sets represent the antagonist effect of the drug-drug interactions. Data represents the average of 5 independent experiments.
(PDF)

S4 Fig The Effect of ‘priming’ with Quercetin prior to cisplatin treatment on cell viability. Cell viability was examined following treatment with quercetin (40 μM), cisplatin (100 μM) and ‘priming’ with quercetin prior to cisplatin treatment. **(A)** MDA-MB231, **(B)** HT-29 **(C)** MCF10A cell viability following treatments. Data represents the average of 3 independent experiments ± SEM. ** p<0.01 *vs* non-treatment, ¢ p<0.05 *vs* Quercetin.
(PDF)
