# Supplementary material for: Withania somnifera Root Extract Enhances Chemotherapy through ‘Priming’
Source: PLoS One. 2017 Jan 27;12(1):e0170917. doi: 10.1371/journal.pone.0170917 (PMC5271386; doi:10.1371/journal.pone.0170917)
Supplement: S1 Fig — HPTLC image graphs of the extract samples. Graph A is measured at the wavelength 366 nm and B is the same graph under white RT light. BP means the extraction method is followed by British Pharmacopoeia. Two replications of each extract samples. The Rf value represents 0.09 (Withaferin A), 0.014 (Standard/Withanolide A) 0.24 (Withanolide B),0.6 (β-sitosteol). (PDF) [file pone.0170917.s001.pdf]

Supporting Information for: *Withania Somnifera* Root Extract Enhances Chemotherapy Through ‘Priming’

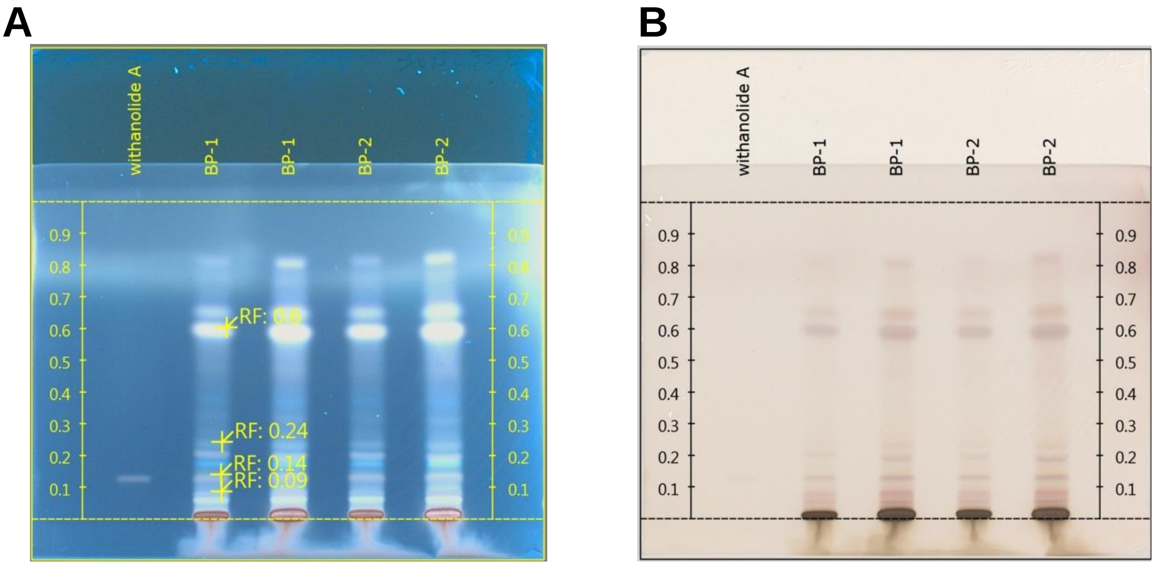

Figure S1. HPTLC. HPTLC image graphs of the extract samples. Graph A is measured at the wavelength 366 nm and B is the same graph under white RT light. BP means the extraction method is followed by British Pharmacopoeia. Two replications of each extract samples. The Rf value represents 0.09 (Withaferin A), 0.014 (Standard/Withanolide A) 0.24 (Withanolide B),0.6 (β-sitosteol).

Henley *et al.,* 2016
