# Supplementary material for: Withania somnifera Root Extract Enhances Chemotherapy through ‘Priming’
Source: PLoS One. 2017 Jan 27;12(1):e0170917. doi: 10.1371/journal.pone.0170917 (PMC5271386; doi:10.1371/journal.pone.0170917)
Supplement: S2 Fig — (A) Ashwagandha root extract total ion chromatogram, (B) Ashwagandha root extract 471 [M+H]+ ion chromatogram, (C) Withanolide, peak 1 mass spectrum, (D) Withanolide, peak 2 mass spectrum, (E) Withanolide, peak 3 mass spectrum. (PDF) [file pone.0170917.s002.pdf]

Supporting Information for: *Withania Somnifera* Root Extract Enhances Chemotherapy Through 'Priming'

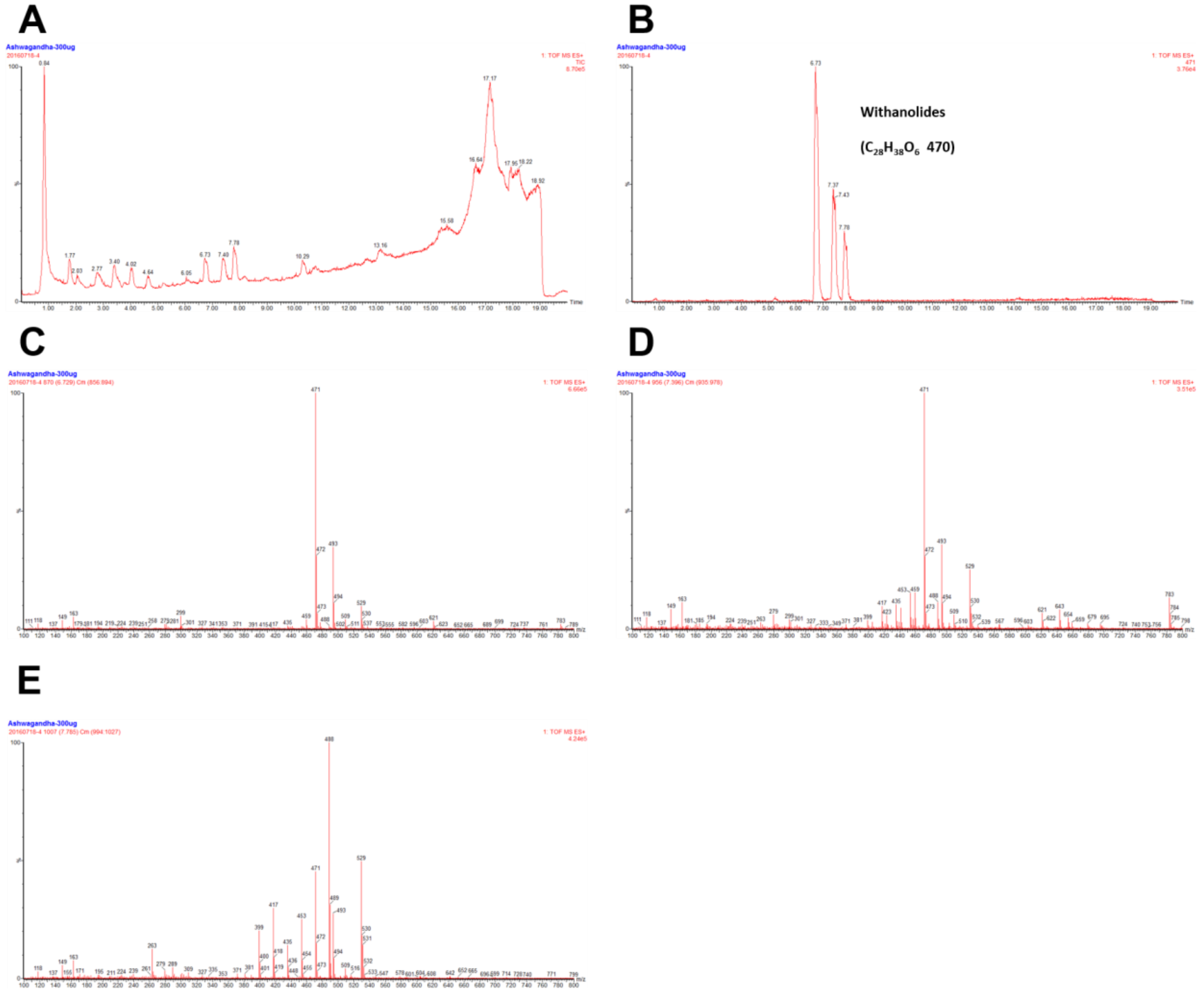

Figure S2. HPLC-MS/MS data (A) Ashwagandha root extract total ion chromatogram, **(B)** Ashwagandha root extract 471 $[M+H]^+$ ion chromatogram, **(C)** Withanolide, peak 1 mass spectrum, **(D)** Withanolide, peak 2 mass spectrum, **(E)** Withanolide, peak 3 mass spectrum.
