# Supplementary material for: Withania somnifera Root Extract Enhances Chemotherapy through ‘Priming’
Source: PLoS One. 2017 Jan 27;12(1):e0170917. doi: 10.1371/journal.pone.0170917 (PMC5271386; doi:10.1371/journal.pone.0170917)
Supplement: S3 Fig — The interactions of W. somnifera and cisplatin was assessed by the methods described in Prichard and Shipman (29). Cell viability was examined following treatment with W. somnifera root extract (0 μg/mL -10 μg/mL) in combination with cisplatin (0 μM -150 μM). (A) MDA-MB231, (B) HT-29 (C) MCF10A 3D models and data table sets represent the antagonist effect of the drug-drug interactions. Data represents the average of 5 independent experiments. (PDF) [file pone.0170917.s003.pdf]

Supporting Information for: *Withania Somnifera* Root Extract Enhances Chemotherapy Through 'Priming'

A MDA-MB231

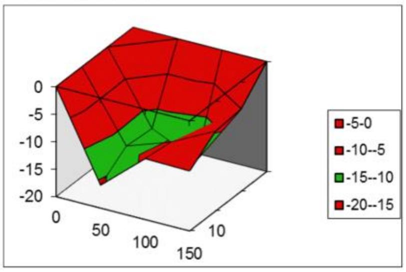

MDA-MB231						
**10**	0	-15.84	-8.95	-9.12	SYNERGY PLOT	
**5**	0	-12.35	-9.56	-5.51	SYNERGY	0
**1**	0	-12.77	-16.43	-5.06	95% CI	NS
**0**	0	0	0	0	ANTAGONISM	-95.59
	**0**	**50**	**100**	**150**	95% CI	-2 - -189

B HT-29

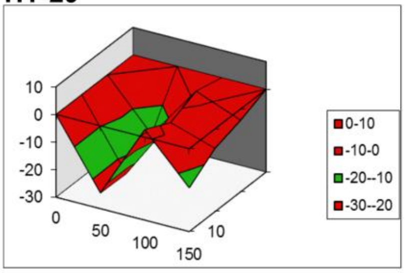

HT-29						
**10**	0	-24.28	2.77	-14.16	SYNERGY PLOT	
**5**	0	-24.48	-1.91	-7.11	SYNERGY	5.09
**1**	0	-17.6	2.32	-4.07	95% CI	NS
**0**	0	0	0	0	ANTAGONISM	-93.61
	**0**	**50**	**100**	**150**	95% CI	0 - -187

C MCF10A

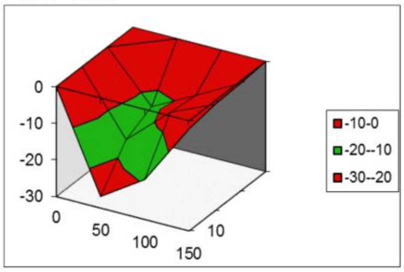

MCF10A						
**10**	0	-26.83	-19.1	-2.21	SYNERGY PLOT	
**5**	0	-16.78	-6.23	-0.97	SYNERGY	0
**1**	0	-13.21	-7.02	-0.65	95% CI	NS
**0**	0	0	0	0	ANTAGONISM	-93
	**0**	**50**	**100**	**150**	95% CI	-13 - -173

Figure S3. Evaluating the drug-drug interactions upon combining treatments of *W. somnifera* and cisplatin. The interactions of *W. somnifera* and cisplatin was assessed by the methods described in Prichard and Shipman (29). Cell viability was examined following treatment with *W. somnifera* root extract (0 µg/mL -10 µg/mL) in combination with cisplatin (0 µM -150 µM). **(A)** MDA-MB231, **(B)** HT-29 **(C)** MCF10A 3D models and data table sets represent the antagonist effect of the drug-drug interactions. Data represents the average of 5 independent experiments.
