# Supplementary material for: Withania somnifera Root Extract Enhances Chemotherapy through ‘Priming’
Source: PLoS One. 2017 Jan 27;12(1):e0170917. doi: 10.1371/journal.pone.0170917 (PMC5271386; doi:10.1371/journal.pone.0170917)
Supplement: S4 Fig — Cell viability was examined following treatment with quercetin (40 μM), cisplatin (100 μM) and ‘priming’ with quercetin prior to cisplatin treatment. (A) MDA-MB231, (B) HT-29 (C) MCF10A cell viability following treatments. Data represents the average of 3 independent experiments ± SEM. ** p<0.01 vs non-treatment, ¢ p<0.05 vs Quercetin. (PDF) [file pone.0170917.s004.pdf]

Supporting Information for: *Withania Somnifera* Root Extract Enhances Chemotherapy Through 'Priming'

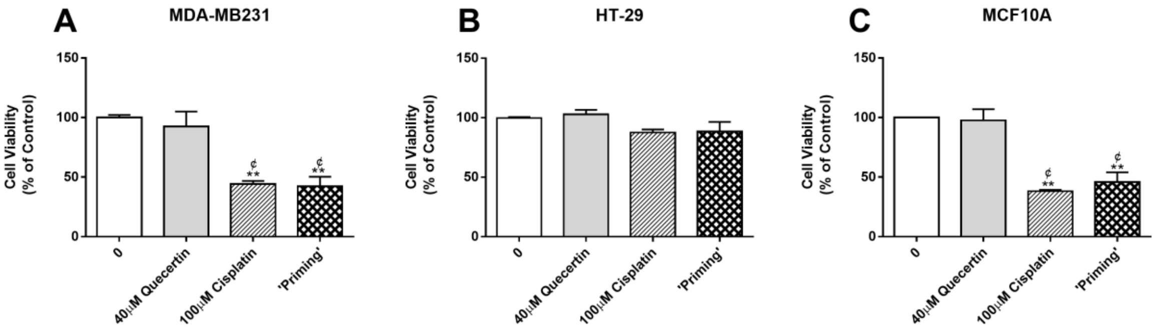

Figure S4. The effect of 'priming' with Quercetin prior to cisplatin treatment on cell viability. Cell viability was examined following treatment with quercetin (40 µM), cisplatin (100 µM) and 'priming' with quercetin prior to cisplatin treatment. **(A)** MDA-MB231, **(B)** HT-29 **(C)** MCF10A cell viability following treatments. Data represents the average of 3 independent experiments ± SEM. ** $p<0.01$ *vs* non-treatment, ¢ $p<0.05$ *vs* Quercetin.

Henley *et al.,* 2016
